# Four clinically utilized drugs were identified and validated for treatment of adrenocortical cancer using quantitative high-throughput screening

**DOI:** 10.1186/1479-5876-10-198

**Published:** 2012-09-21

**Authors:** Naris Nilubol, Lisa Zhang, Min Shen, Ya-Qin Zhang, Mei He, Christopher P Austin, Electron Kebebew

**Affiliations:** 1 Endocrine Oncology Branch, Center for Cancer Research, National Cancer Institute, NIH, Rm. 3-3940, 10 Center Drive, MSC 1201, Bethesda, MD, 20892, USA; 2 National Center for Advancing Translational Sciences, NIH Chemical Genomics Center, NIH, Bethesda, MD, USA

**Keywords:** Adrenocortical cancer, High throughput drug screening, Chemotherapy, Drug repurposing, Indication switching

## Abstract

**Background:**

Drug repurposing for cancer treatment is an emerging approach to discover clinically approved drugs that demonstrate antineoplastic effect. The effective therapeutics for patients with advanced adrenocortical carcinoma(ACC) are greatly needed. The objective of this study was to identify and validate drugs with antineoplastic effect in ACC cells using a novel quantitative high-throughput drug screening (qHTS) technique.

**Methods:**

A quantitative high-throughput proliferation assay of 2,816 clinically approved drugs was performed in the NCI-H295R ACC cell line. We validated the antiproliferative effect of candidate compounds in NCI-H295R cells. Further validation was performed in 3-dimensional multicellular aggregates (MCA) of NCI-H295R and SW-13 cell lines.

**Results:**

We identified 79 active compounds against ACC cells; 21 had an efficacy ≥60% and IC_50_ <1 μM. The top drug categories enriched were cardiotonic, antiseptic, and antineoplastic. We selected Bortezomib, ouabain, Methotrexate, pyrimethamine for validation. All had an antiproliferative effect in monolayer culture of NCI-H295R cells at clinical achievable serum level. Bortezomib and ouabain inhibited growth of MCA in both cell lines at a low concentration (10 fold below IC_50_). Methotrexate inhibited growth and caused disintegration of MCA in both cell lines at concentrations well below the maximum serum level (10 to 100 fold of IC_50_). Pyrimethamine caused growth inhibition in both cell lines at 10 fold of IC_50_ concentration.

**Conclusions:**

qHTS of previously approved compounds is an effective and efficient method to identify anticancer drugs for a rare cancer such as ACC. We have validated the antineoplastic effect of Bortezomib, ouabain, Methotrexate and pyrimethamine, which could be translated into clinical trials in patients with locally advanced and/or metastatic ACC.

## Background

Adrenocortical cancer (ACC) is a rare and aggressive malignancy. Patients with ACC generally have a dismal prognosis, with only 16% to 38% 5-year survival rate [1,2]. While surgical resection may cure some patients with localized disease, most patients present with advanced disease or develop local recurrence and distant metastasis after surgery [3]. Five-year disease specific survival varies depending on several prognostic factors, ranging from 9% to 60% [3,4]. Unfortunately, there is no effective therapy that provides a durable objective response in patients with advanced and metastatic ACC.

Although there has been an increased focus on personalized treatment in the post-genomic era, this approach is in its infancy for ACC as the genetic causes are poorly understood. The cost and time needed for developing a new therapy for rare cancers are also often prohibitive. An alternative approach to cancer therapy that is just beginning to be explored is the exploitation of established drugs that have already been approved for clinical use for one or more indications and with known toxicity. Drug “repositioning”, “repurposing” or “indication switching” has several important advantages that circumvent other traditional drug discovery approaches [5-7]. Since the pharmacokinetic, pharmacodynamic, and toxicity profiles of the drugs are well known, an immediate translation into phase II or III clinical trial to test the efficacy can be performed. In addition, potential new mechanisms of drug action against tumor cells or biological process involved in carcinogenesis can be further explored. Furthermore, some drugs found to have an anticancer effect may reduce the risk of developing cancer and thus could be used for prevention of common cancers in high risk individuals [8-11]. Given these inherent advantages of drug repurposing and the lack of effective therapy for ACC, we performed quantitative high throughput screening (qHTS) of 2,816 clinical approved drugs in the NCI-H295R ACC cell line. We then validated a select number of drugs in 3-dimensional multicellular aggregates (MCA) of NCI-H295R and SW-13 ACC cell lines.

## Materials and methods

### ACC cell culture

NCI-H295R cells and SW-13 cells were both grown and maintained in Dulbecco modified Eagle medium (DMEM) supplemented with 0.1% premix ITS^+^ (BD Biosciences, San Jose, CA), and serum (Nu-Serum I [2.5%], BD Biosciences, San Jose, CA) in a standard humidified incubator at 37°C in a 5% CO_2_ atmosphere. Cells were routinely sub-cultured every 3 to 4 days. The establishment and characterization of NCI-H295R and SW-13 cell lines were described by Gazdar et al. [12] and Leibovitz et al. [13], respectively. NCI-H295 cells originated from adrenal cortex of a 48 year-old African American female patient with adrenocortical carcinoma. NCI-H295R was adapted from the NCI-H295 pluripotent ACC cell line established by Gazdar et al [12]. The original cells were adapted to a culture medium which resulted in a decreased population doubling time from 5 days to 2 days. While the original cells grew in suspension, the adapted cells were selected to grow in a monolayer. This cell line retains the ability to produce adrenal androgens, aldosterone and cortisol. It is responsive to angiotensin II and potassium ions. SW-13 cells originated from adrenal cortex of a 55 year-old Caucasian female with metastatic primary small cell adrenal carcinoma [13]. Itoh et al. demonstrated an increase production of dehydroepiandrosterone sulfate (DHEA-S) by angiotensin-II administration in SW-13 cell line, consistent with steroidogenesis in human adrenal cortex [14]. However, SW-13 cells do not produce cortisol and only produce very small amount of aldosterone [15].

Both cell lines were purchased from American Type Tissue Collection™ (Manassas, VA) and authenticated by Short Random Repeat profiling. The Short Random Repeat profiles are the product of polymerase chain reaction using the commercially available Promega PowerPlex 1.2 kit (Promega North America, Madison, WI) with AmpliTaq Gold polymerase (Applied Bios stems, Foster City, CA) and 1 ng of template DNA. Our NCI-H295R cells used in initial high throughput screening process were in their 5^th^ passage from original cells received from the American Type Tissue Collection™. We subsequently confirmed cortisol production by ELISA in our NCI-H295R cell line in later passages.

### National institutes of health chemical genomic center (NCGC) pharmaceutical library screening

The NCGC Pharmaceutical Collection (NPC) consists of 2,816 small molecule compounds with 52% of the drugs approved for human or animal use by the United States Food and Drug Administration (FDA) The list of 2,816 compounds is provided in Additional file 1. The remaining drugs are either approved for use in other countries, such as Europe, Canada, or Japan, or are compounds that have been tested in clinical trials. Additional detailed information on the drug library can be found at http://tripod.nih.gov/npc/.

The compounds from NPC library were prepared as 15 interpolate titrations, which were serially diluted 1:2.236 in dimethyl sulfoxide (DMSO) (Thermo Fisher Scientific, Waltham, MA) in 384-well plates. The stock concentrations of the test compounds ranged from 10 mM to 0.13 μM. The transfer of the diluted compounds from 384-well plates to 1536-well plates was performed using an Evolution P^3^ system (PerkinElmer Life and Analytical Sciences, Waltham, MA). Each treatment plate included concurrent DMSO, top concentrated positive control wells and concentration-response titrations of positive controls, all occupying columns 1 to 4. Each compound was tested in duplicates. During screening, the compound plates were thawed, sealed and kept at room temperature. After NCI-H295R cells were treated, cells were kept in the incubator for 48 hours.

### Quantitative high throughput proliferation assay

Cell viability after drug treatment was measured using a luciferase-coupled ATP quantization assay (CellTiter-Glo®, Promega, Madison, WI) in NCI-H295R cells. The change of intracellular ATP content indicates the number of metabolically competent cells after drug treatment. NCI-H295R cells were harvested from T225 flask and resuspended in ITS supplemented DMEM serum free medium at a concentration of 300,000 cells/ml. Then 5 μl of resuspended cells was dispensed into each well of white, solid bottom, 1536-well tissue culture–treated plates using a Multidrug Combo dispenser. After overnight culture at 37°C with 5% CO_2_, a total of 23 nl of compounds at 8 selected concentrations from the NPC or positive control (10 mM stock of doxorubicin hydrochloride) in DMSO was transferred to each well of the assay plate using a pin tool (Kalypsys, San Diego, CA). The final concentration of the compounds in the 5 μl assay volume ranged from 0.6nM to 46 μM with a 1:5 dilution factor.

**Figure 1:**
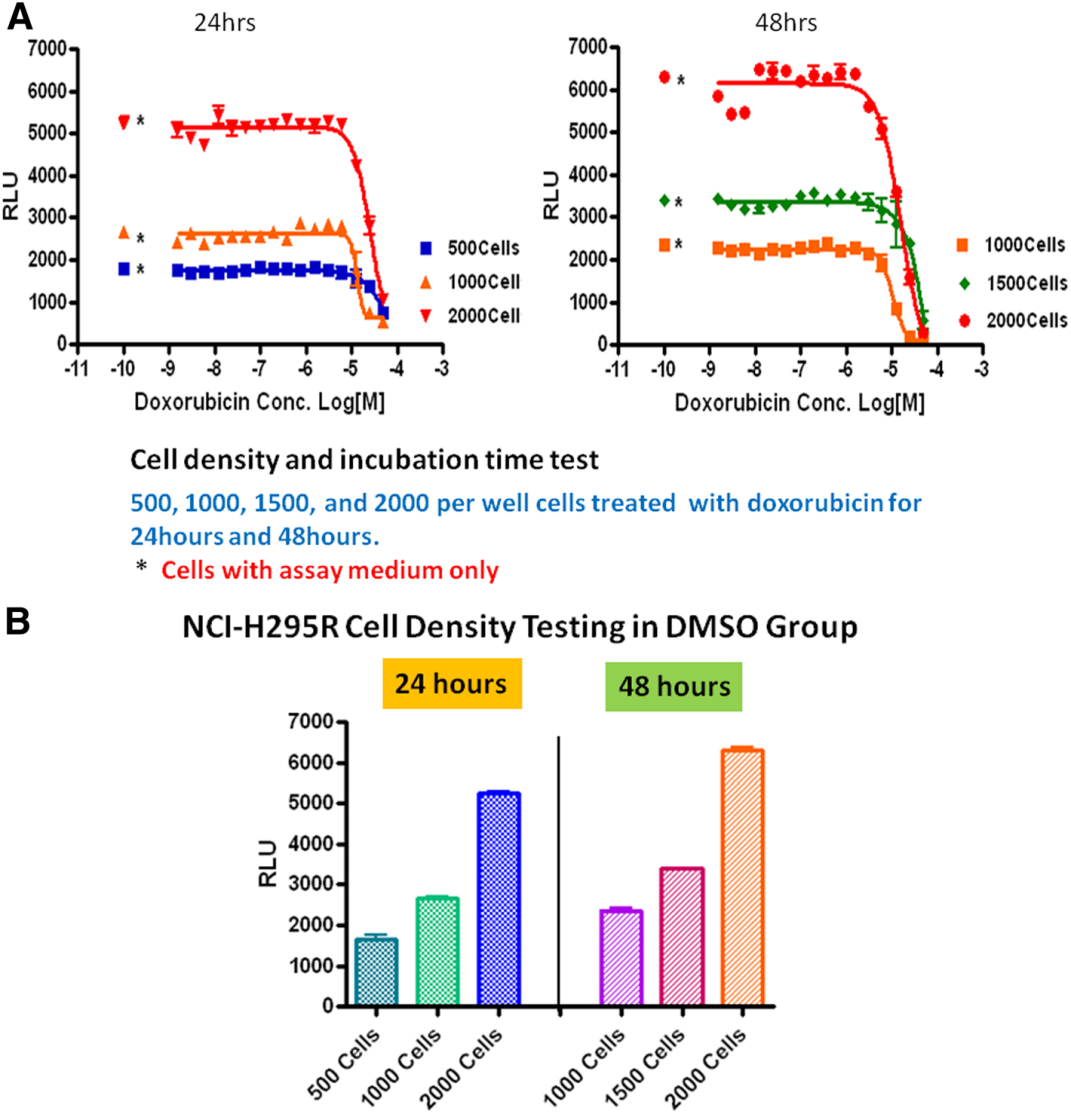
**A) NCI-H295R cell density testing for quantitative high throughput screening.** At 1,500 cells per well, the optimal dose–response curve was observed. **B**) At 2,000 cells per well, cellular proliferation was observed in DMSO treated NCI-H295R cells.

We performed cell density testing by seeding 500, 1000, 1500, and 2000 cells per well. The cell density that provided optimal dose–response curve was 1500 cells per well (Figure 1A). At 2,000 cells per well, cellular proliferation was observed in vehicle control group (DMSO) at 48 hours, suggesting sufficient growth condition (Figure 1B). The plates were further incubated at 37°C and 5% CO_2_ for 48 hours. Then 4 μl of CellTilter-Glo®luminescent substrate mix (Promega, Madison, WI) was added to each well. The plate was incubated at room temperature for 20 minutes. A ViewLux plate reader (PerkinElmer, Waltham, MA) with clear filter was used to measure the number of metabolically competent cells. The experiment was performed once.

To determine compound activity in the qHTS assay, the titration-response data for each sample was plotted and modeled by a four parameter logistic fit yielding IC50 and efficacy (maximal response) values. Raw plate reads for each titration point were first normalized relative to positive control (doxorubicin hydrochloride, 100% inhibition) and DMSO only wells (basal, 0%). The screening performance was evaluated by 1) coefficient of variation (CV) defined as the standard deviation of compound area (the area from the 5^th^ to 48^th^ column in 1536-well plate where the compounds from NCGC pharmaceutical library are plated and tested) to the mean of compound area. The acceptance criterion is met when the CV of each signal is ≤ 20% for DSMO and low concentration plates, high concentration plates (plate # 9, 10, 19 and 20) in Additional file 2: Figure S1 were not applied, 2) signal-to-background (S/B) ratio. The S/B ratio must be >2. 3) Z-factor which is a measure of statistical effect size as described by Zhang et al [16]. In brief, Z = 1 is an ideal assay where no variation (standard deviation = 0) or dynamic range is infinite, Z between 0.5 and 1.0 is an excellent assay, Z between 0 and 0.5 is a marginal assay, and Z < 0 is not a useful assay.

We selected the candidate compounds that demonstrated antiproliferative efficacy of > 60% and IC_50_ < 1 μM for further validation. Compounds that meet our criteria are likely to have antineoplastic activity against ACC at clinically achievable serum level. We then analyzed the characteristics of these drugs, including the current available preparation and route of administration, maximum serum concentrations after systemic delivery, and drug half-life in humans (Table 1). We selected 4 active drugs that could be administered orally or intravenously with IC_50_ equal to or below maximal serum concentration for validation. To further assess the drug categories that were active against NCI-H295R, we performed enrichment analysis by therapeutic category. The enrichment score is the ratio of number of active drugs to the total of numbers of tested drugs in the same therapeutic category.

**Table 1:** **Characteristics of 21 active compounds**^**1**^**against NCI-H295R cells with efficacy higher than 60% and potency below 0.9 μM**

**Drug**	**Route**	**IC**_**50**_**(μM)**	**Max serum level (μM)**	**Elimination half life**	**Efficacy**	**Mode of action**
***Aclarubicin***	IV	0.94	0.34	13.3 hrs	-122.30	Topoisomerase I and II inhibitor
***Actinomycin D***	IV	0.38	0.02-0.08	36 hrs	-112.51	Inhibitors of DNA synthesis
***Bortezomib***	IV	0.34	0.16-0.31	9-15 hrs	-77.11	Proteasome inhibitor;
***Carboquone***	PO/IV	0.75	0.62-0.74 (200-240 ng/mL)	0.5 hrs	-119.79	Alkylating agents
***Ciclopirox***	Topical	0.64	N/A - topical only	2.2 hrs in rabbits	-61.11	Chelation of polyvalent metal cations
***Deslanoside***		0.34	0.16	51 hrs	-110.35	Na+/K + ATPase inhibition;
***Digitoxin***	IV	0.08	0.05-0.2	4-9 days	-109.13	Na+/K + ATPase inhibition;
***Digoxin***	PO/IV	0.19	0.003	36-48 hrs	-111.40	Na+/K + ATPase inhibition;
***Ecteinascidin***	IV	0.01	Up to 0.05	50-180 hrs	-116.82	Disruption of DNA strand
***Homoharringtonine***	SQ	0.21	0.176	9.3 hrs	-125.33	Protein synthesis inhibitor
***Lanatoside A***	IV/PO	0.19	**N/A**	**N/A**	-114.10	Na+/K + ATPase inhibition;
***Lanatoside C***	IV/PO	0.3	**N/A**	**N/A**	-117.82	Na+/K + ATPase inhibition;
***Methotrexate***	PO/IV	0.02	10-100	8-15 hrs	-74.91	DHFR inhibitor
***Metildigoxin***	PO/IV	0.24	**N/A**	**N/A**	-110.90	Na+/K + ATPase inhibition;
***Niclosamide***	PO/IV	0.27	**N/A**	**N/A**	-94.29	Uncouple oxidative phosphorylation
***Ouabain***	IV	0.08	0.128	18 hrs	-110.64	Na+/K + ATPase inhibition;
***Plicamycin***	IV	0.94	0.6 μM	10.5 hrs	-117.62	RNA synthesis inhibitor;
***Proscillaridin A***	IV/PO	0.01	0.019 (IV); 0.0019 (PO)	23-33 hrs	-110.55	Na+/K + ATPase inhibition;
***Pyrimethamine***	PO/IV	0.75	8.04	140-190 hrs	-81.43	DHFR inhibitor
***Rotenone***	N/A	0.75	**N/A**	**N/A**	-69.22	Electron transport chain in mitochondria interference
***Trimetrexate glucuronate***	IV	0.01	N/A	11-16 hrs	-80.39	DHFR inhibitor

^1^The references for this table are provided in Additional file 3.

N/A: no available data.

**Figure 2:**
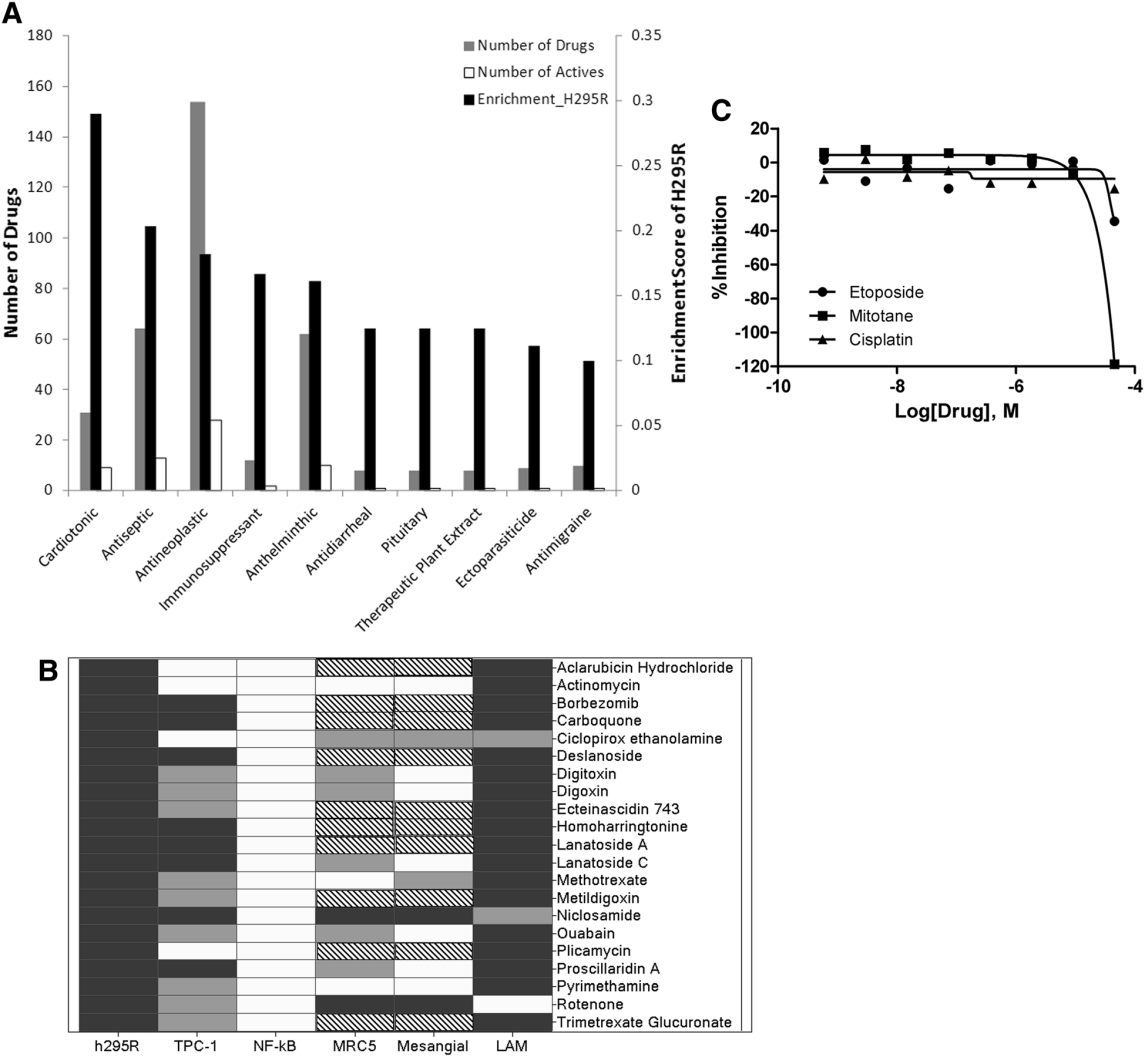
**A) Enrichment analysis of screened active compounds by therapeutic category.B**) Drug activity profile across different cell lines and primary cell culture of 21 active drugs in H295R cells. Black box indicates active compound, grey box indicates compound with inconclusive activity, white box represents inactive compounds, and the compounds that were not tested are labeled in stripe. The criteria for compound activity are described in Data Analysis section. TPC-1: papillary thyroid cancer cell line, NF-kB: ME-180 human cervical carcinoma cell line, MRC5: normal human fetal lung fibroblasts, Mesangial: human kidney glomerular mesangial cell line, LAM: lymphoangioleiomyosis cell line (LAM, a rare lung disease that results in a proliferation of disorderly smooth muscle growth). **C**) Dose–response curve of NCI-H295R treated with cisplatinum, etoposide and mitotane.

We explored the use of qHTS as a tool to screen for compounds with antiproliferative activity in normal cells or in other cancer cells by assessing the activities of active drugs in the following normal and cancer cell lines: TPC-1: papillary thyroid cancer cell line, NF-kB: ME-180 human cervical carcinoma cell line, MRC5: normal human fetal lung fibroblasts, Mesangial: human kidney glomerular mesangial cell line, LAM: lymphoangioleiomyosis cell line (LAM, a rare lung disease that results in a proliferation of disorderly smooth muscle growth) (Figure 2B).

### *In vitro* validation of qHTS assay

1.Cell proliferation assay

The candidate drugs with antiproliferative activity were validated in NCI-H295R ACC cells using the CyQuant® Cell Proliferation Assay (Invitrogen^TM^ Corp., Carlsbad, CA). NCI-H295R (6 × 10^3^) cells were plated into 96-well clear bottom, black plate (Costar®, Corning, NY). Each well contained 100 μl of culture media with serum. After 24 hours (Day 0), 100 μl of fresh culture media containing double concentrations of the indicated drugs or corresponding vehicles was added into each well. Additional 100 μl of fresh media containing the 1× concentration of the tested drugs or vehicles were added 48 hours (Day 2) after initial administration to support optimal cell growth. We treated the cells with 3 concentrations of each selected drugs as well as their respective vehicle controls based on their half maximal inhibitory concentration (IC_50_) from the qHTS. The three concentrations used were 10 fold of IC_50_, IC_50_, and 0.1 fold of IC_50_. Each concentration was performed in quadruplicates. CyQuant® Cell Proliferation Assay (Invitrogen^TM^ Corp., Carlsbad, CA) was performed on Day 0, 1, 2, 3 and 4 according to the manufacturer’s protocol. The cell densities in the 96-well black plates were determined using a SpectraMax M5e 96-well fluorescence micro plate reader (Molecular Devices, Sunnyvale, CA) at 485 nm/538 nm. For each drug tested, the experiments were repeated at least three times. We selected the concentrations of the drugs that demonstrated antiproliferative effect in NCI-H295R cells and validated the in SW-13 cells. SW-13 (4 × 10^3^) cells were plated into 96-well plate and tested using the same cell proliferation assay protocol.

2.*In vitro* validation of candidate drugs in 3-dimensional multicellular aggregates (MCA).

Further validation tests of the candidate drugs were performed on MCA of NCI-H295R and SW-13 cells. Although monolayer cell cultures can provide cell-specific response to drugs, this model, however, lacks the microenvironment of 3-dimentional solid tumors observed *in vivo*, such as hypoxic tissue areas, regions of differential growth and cell cycling, as well as poor availability of delivered drugs in deeper tumor tissue layers, which can be found in MCA [17].

MCA of NCI-H295R cells form compact tumor spheroids in suspension culture condition. We generated NCI-H295R tumor spheroids by plating 1.0 × 10^5^cells/well into Ultra Low Cluster, 24-well plate (Costar®, Corning, NY) and incubated the cells at 37°C in 5% CO_2_ for two weeks. The media was changed twice a week. At the beginning of the 3^rd^ week of culture, NCI-H295R MCA were treated with selected drugs and their corresponding vehicle controls using concentrations based on IC_50_ from the monolayer proliferation assay and maximal serum levels (Table 2).

**Table 2:** **Drug concentrations tested in NCI-H295R and SW-13 MCA**^**1**^

**Drugs**	**IC**_**50**_^**2**^**(μM)**	**Maximum serum level (μM)**	**Conc**^**3**^**. tested in MCA (μM)**	**Ratio of tested conc. to IC**_**50**_
Bortezomib	0.34	0.31	3.4, 0.34, 0.17, 0.03	10x, 1x, 0.5x, 0.1x
Ouabain	0.08	0.13	0.8, 0.16, 0.08	10x, 2x, 1x
Methotrexate	0.02	10-100	2, 1, 0.2	100x, 50x, 10x
Pyrimethamine	0.75	8.0	7.5, 3.7	10x, 5x

^1^MCA: 3-dimensional multicellular aggregates.

^2^IC_50_: half maximal inhibitory concentration.

^3^Conc: concentrations.

SW-13 cells form 3-dimensional cell growth with the cell aggregates were not as tight and compact as MCA formed by NCI-H295R. We utilized the same protocol described above to grow SW-13 MCA. SW-13 MCA were treated with selected drugs at the beginning of the 4^th^ week of culture to allow the cells to form denser aggregates. All the concentrations of selected drugs and their corresponding vehicle controls were identical to those used in NCI-H295R MCA (Table 2).

All experiments were performed in triplicates for each testing concentration. SW-13 and H295R MCA were continuously treated for 6 and 10 weeks, respectively. MCA were photographed with Nikon coolpix 990 with Nikon MDC lens (Nikon, Inc, Melleville, NY) under 12.5× magnification microscope (Olympus SZX9 microscope with DF Plapo 1X-2 lens, Olympus America, Inc, Center Valley, PA). The experiments were repeated at least twice. A quantitative analysis of MCA was performed by measuring the area of MCAs using Image software (National Institutes of Health, Bethesda, Maryland, USA, http://rsb.info.nih.gov/ij/, 1997-2008).

### Caspase-3 and -7 activation assay

The effect of Bortezomib, ouabain, Methotrexate and pyrimethamine on caspase 3 and 7 activity was evaluated using the Caspase-Glo® 3/7 kit (Promega North America, Madison, WI) according to the instructions provided by the manufacturer. Briefly, NCI-H295R cells (6 × 10^3^ cells/well in 100 μl) were placed in a 96-well white walled clear bottom plate (Lonza, Allendale, NJ). Cells were incubated for 24 hours then treated with 1) 0.34 μM Bortezomib (IC50), 2) 0.08 μM ouabain (IC50), 3) 0.2 μM Methotrexate (10-fold of IC50), and 4) 7.5 μM pyrimethamine (10-fold of IC50) and their respective vehicle controls.

The Caspase-Glo® 3/7 reagent (100 μl/well) was added at 48 and 72 hours post-treatment. The plates were incubated in the dark for 60 minutes at room temperature on plate shaker. Luminescence was measured in SpectraMax M5e plate reader (Molecular Devices, Sunnyvale, CA). All the treatments were performed in quadruplicates. The caspase activity of treated NCI-H295R cells was compared to vehicle controls. Data are presented as mean ratio of caspase activity in treated cells to vehicle controls ± SD.

### Cell cycle analysis

SW-13 and NCI-H295R cells were plated in 75 cm^2^ tissue culture flasks at 5 × 10^5^ and 1 × 10^6^ cells per flask, respectively. Cells were incubated for 24 hours, culture medium was removed, and 15 ml fresh culture medium containing vehicle controls or fresh medium with 200 nM Methotrexate or 7.5 μM pyrimethamine was added into each flask. Three days later, cells were harvested, washed, resuspended with PBS, and fixed with ice-cold 70% ethanol at 4°C. After washing with PBS, rib nuclease A was added to the cell suspension and incubated at 37°C for 30 min. Propidium iodide (PI) (50 mg/ml in PBS) was added, and samples were stored at 4°C until analysis. Flow cytometry analysis for cell cycle was performed on a FACScan using CellQuest software (BD Biosciences, San Jose, CA). Data files were generated for more than 20,000 events (cells) per sample gated on singlet cells. Cell clumps, debris, and doublets were excluded by PI fluorescence pulse width and pulse area measurements. Cell cycle analysis on the gated PI distribution was performed using Modify software (Verity Software House, Inc., Topsham, ME).

#### Data analysis

For the high throughput screening assay, the titration-response data of each sample were plotted and modeled by a four-parameter logistic fit to determine compound activity [18]. Curve-fits were then classified by criteria previously described [18]. In brief, Class 1.1 and 1.2 were full curves containing upper and lower asymptotes with efficacy ≥ 80% and < 80%, respectively. Class 2.1 and 2.2 were incomplete curves having only one asymptote with efficacy ≥ 80% and < 80%, respectively. Class 3 curves showed activity at only the highest concentration or were poorly fit. Class 4 curves were inactive having a curve-fit of insufficient efficacy or lacking a fit altogether. Only compounds that demonstrated Class 1.1, 1.2, 2.1, and 2.2 with the maximal inhibition over 60% were considered active against NCI-H295R cells. Compounds that demonstrated Class 2.2 with the maximal inhibition ≤60% or Class 3 curve were considered inconclusive with low confidence activity.

Cell proliferation assay was analyzed using the paired *t* test to determine the statistical difference between the density of treated cells and corresponding vehicle control. The area of MCA was compared using the paired *t* test. The two-sided *p* value less than 0.05 was considered statistically significant.

## Results

### Quantitative high throughput screening of clinical drug library

Overall performance of qHTS was excellent with low variation, high S/B ratio and Z-factor between 0.5 to 1. The screening performance and plate variation are summarized in Additional file 2: Figure S1. The quantitative high throughput screening identified 79 high-confidence, active compounds (Class 1.1, 1.2, 2.1, and 2.2 with the maximal inhibition over 60%) in the NCI-H295R cell line (Additional file 4: Table S1.). Twenty one of these compounds had efficacy higher than 60% and potency (IC_50_) below 1 μM. These 21 compounds were distributed across various therapeutic categories and mode of actions (Table 1). Top 5 drug categories active in the NCI-H295R cells (enrichment ratio >15%) demonstrated by the enrichment analysis were cardiotonic, antiseptic, antineoplastic, immunosuppressant, and anthelmintic (Figure 2A). Eight of 21 active compounds with >60% efficacy were cardiac glycosides (Table 1). The antiproliferative activity of the 21 active compounds against NCI-H295R cells was further analyzed to evaluate the cross activity in other types of normal and cancer cell lines. To evaluate the concept that qHTS can be used to screen drug toxicities in normal cells to guide compound selection, we tested 11 of 21 compounds with activity against NCI-H295R cells in MRC5 and mesangial cells and found that eight showed no antiproliferative or low-confidence activity, which suggests that these drugs had specific antiproliferative activity in particular normal and tumor cells. When compared to other cancer types, a distinct activity pattern was also observed for NCI-H295R cells, in which several of the drugs were also active against other cancer cells, but they usually showed less activity or even no antiproliferative effect in cervical cancer cells (NF-kB) (Figure 2B).

### Validation of candidate active compounds

Based on our selection criteria, 4 promising drugs (Bortezomib, ouabain, methotrexate and pyrimethamine) were chosen for validation (Table 2). All had IC_50_ equal to or below maximum serum concentration in human with antiproliferative efficacy >60%.

Drugs that are known to induce tumor response clinically such as mitotane, cisplatinum and etoposide were in our qHTS drug library [19]. Cisplatinum was not active against NCI-H295R. Mitotane and etoposide only showed antiproliferative effect at high concentration with single point activity (class 3 curve). Dose–response curve of cisplatinum, mitotane and etoposide are shown in Figure 2C.

**Figure 3:**
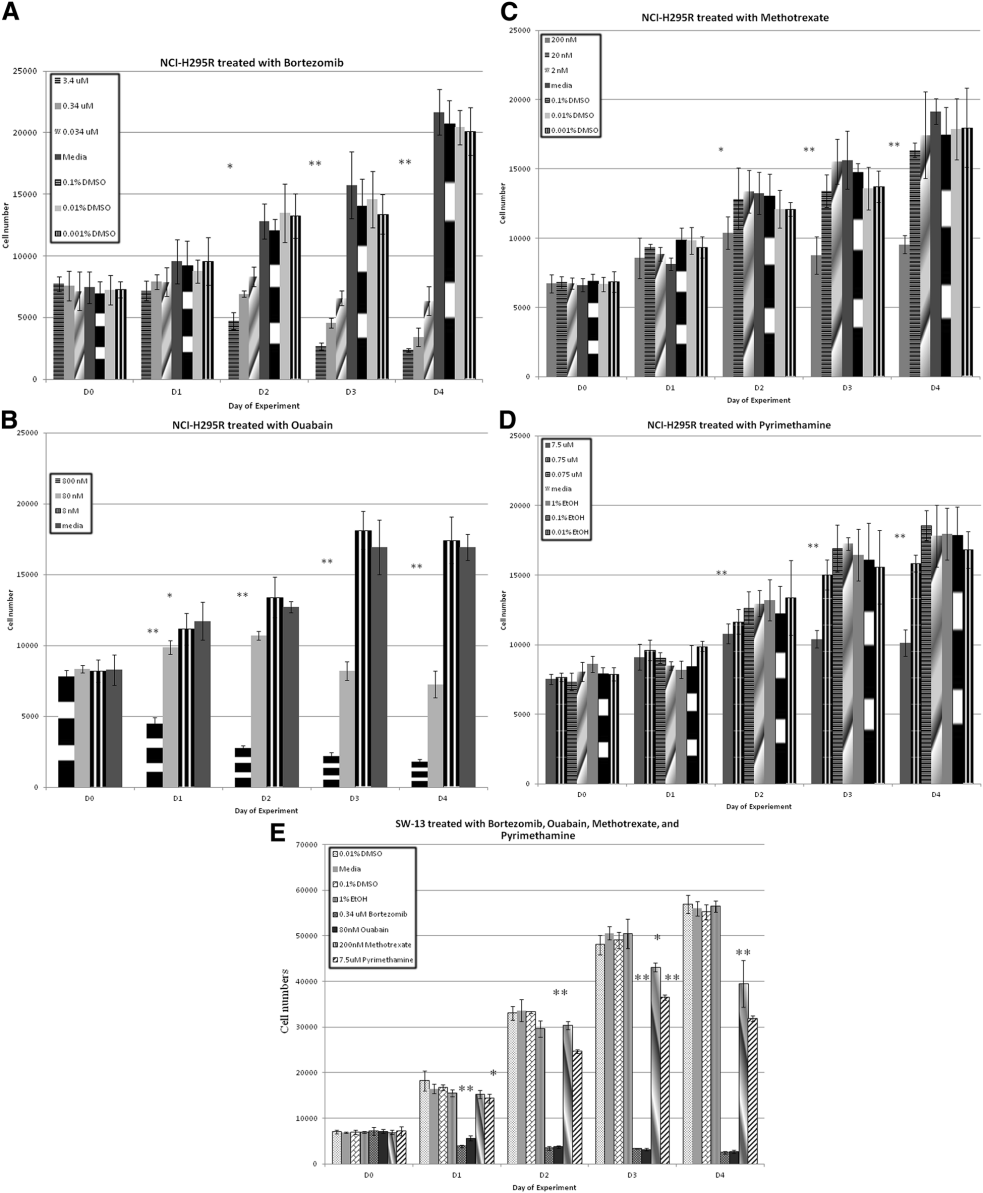
**Cell proliferation assay of NCI-H295R treated with bortezomib (A), ouabain (B), methotrexate (C), and pyrimethamine (D).** Selected effective concentrations of bortezomib, ouabain, methotrexate, and pyrimethamine from cell proliferation assay of NCI-H295R were validated in SW-13 (**E**). The error bar indicates the ranges of cell numbers. * *p* value <0.05 but ≥ 0.01, ** *p* value < 0.01.

We validated the antiproliferative effect of these 4 drugs from the qHTS in NCI-H295R monolayer cell culture. Bortezomib is the first FDA-approved proteasome inhibitor for treatment of mantle cell lymphoma and multiple myeloma. Bortezomib demonstrated remarkable effect on cellular proliferation at concentrations as low as 34 nM (0.1 fold of IC_50_) (Figure 3A). At 34 nM, Bortezomib not only result in 68% growth inhibition, but also caused cell death 11%. The growth inhibition effect was dose-dependent (340 nM and 3.4 μM) (Figure 3A). At 340nM (IC_50_), Bortezomib inhibited cellular proliferation of SW-13 cells by 65% at day 4 (Figure 3E).

**Figure 4:**
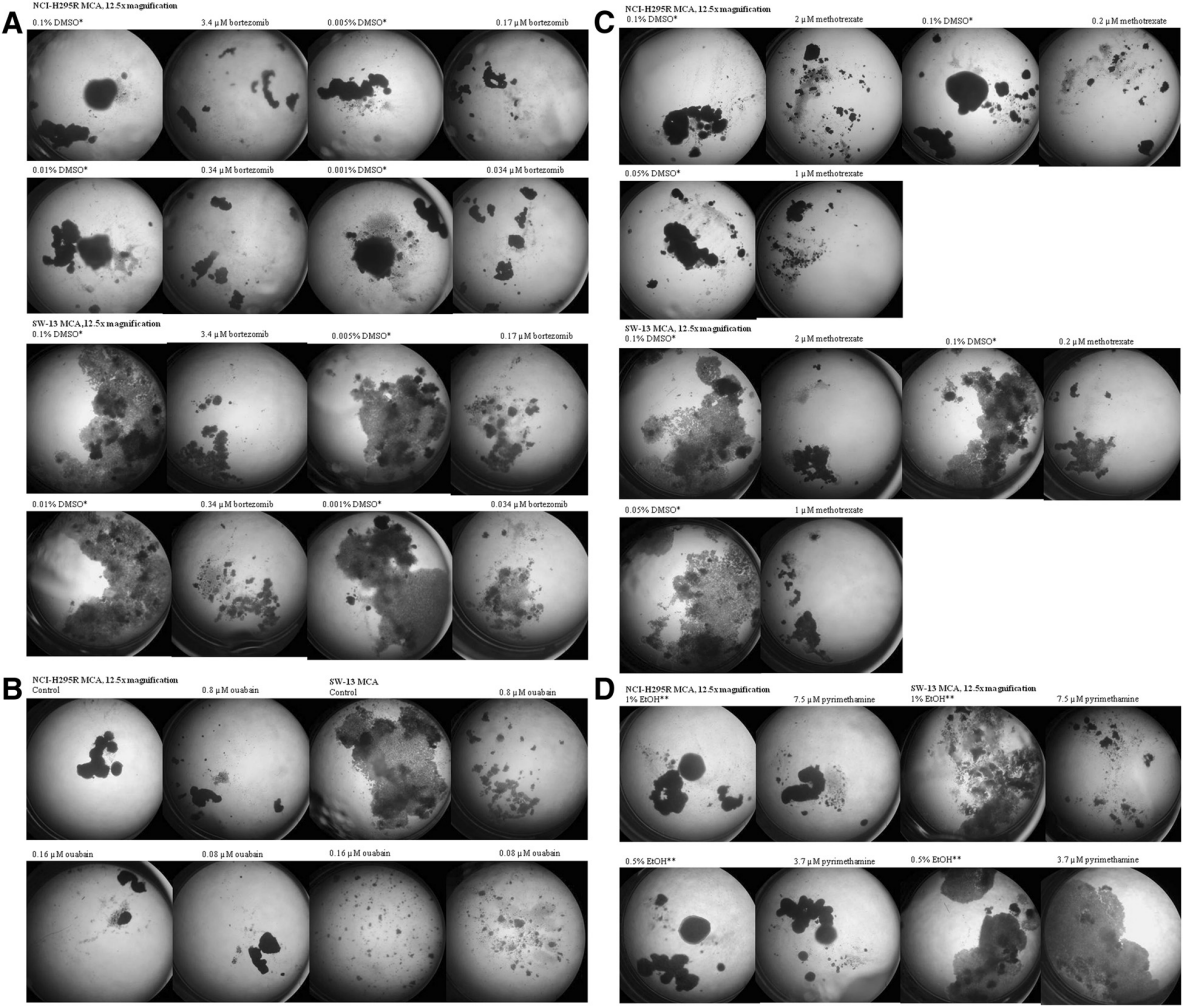
**Three-dimensional multicellular aggregates (MCA) of NCI-H295R and SW13 treated with bortezomib (4A), ouabain (4B), methotrexate (4C), and pyrimethamine (4D).** Cells treated with bortezomib, ouabain, and methotrexate had noticeably smaller MCAs or disintegration of MCAs. Pyrimethamine treated SW13 cells developed noticeably smaller MCAs. Pyrimethamine had no effect on NCI-H295R MCAs.

Once the antiproliferative effect of Bortezomib was confirmed in monolayer cell culture, we tested the effect of Bortezomib in both NCI-H295R and SW-13 MCA. MCA of NCI-H295R and SW-13 cells treated with Bortezomib were significantly smaller at all concentrations (Figure 4A and Table 3).

**Table 3:** The area of NCI-H295R and SW-13 MCAs treated with various concentrations of bortezomib, oubain, methotrexate and 7.5 μM pyrimethamine was significantly smaller than respective controls

**Drugs and concentrations tested in (ratio to IC**_**50**_^**2**^_**)**_		**Area of NCI-H295R MCAs (Mean pixels ± SD)**	***p*-value**	**Area of SW-13 MCAs (Mean pixels ± SD)**	***p*-value**
Bortezomib	0.1% DMSO	2.3 ± 0.7x10^5^	0.02	8.2 ± 2.4x10^5^	0.03
	3.4 μM 0(10x),	10.7 ± 0.8x10^5^		1.0 ± 0.3x10^5^	
	0.01% DMSO	2.6 ± 0.9x10^5^	0.048	1.1 ± 0.2x10^6^	0.02
	0.34 μM (1x),	1.3 ± 0.2x10^5^		1.8 ± 0.2x10^5^	
	0.005% DMSO	2.3 ± 0.5x10^5^	0.01	8.3 ± 1.7x10^5^	0.015
	0.17 μM (0.5x)	1.3 ± 0.2x10^5^		1.0 ± 0.2x10^5^	
	0.001% DMSO	3.0 ± 0.6x10^5^	<0.01	7.5 ± 1.2x10^5^	0.01
	0.03 μM (0.1x)	1.6 ± 0.1x10^5^		5.0 ± 1.6x10^4^	
Ouabain	Media only	2.4 ± 0.07x10^5^	0.01	1.0 ± 0.2x10^6^	0.01
	0.8 μM (10x)	1.5 ± 0.1x10^5^		1.0 ± 0.2x10^5^	
	0.16 μM (2x)	1.6 ± 0.2x10^5^	0.03	1.4 ± 0.1x10^5^	0.01
	0.08 μM (1x)	1.6 ± 0.2x10^5^	<0.01	1.1 ± 0.7x10^5^	<0.01
Methotrexate	0.1% DMSO	2.0 ± 0.2x10^5^	<0.01	7.8 ± 0.5x10^5^	<0.01
	2 μM (100x)	2.3 ± 0.3x10^4^		1.5 ± 0.7x10^5^	
	0.05% DMSO	1.9 ± 0.6x10^5^	0.037	6.7 ± 2.0x10^5^	0.04
	1 μM (50x)	2.0 ± 0.3x10^4^		1.3 ± 0.4x10^5^	
	0.1% DMSO	4.3 ± 1.4x10^5^	<0.01	9.2 ± 0.07x10^5^	0.01
	0.2 μM (10x)	6.1 ± 4x10^4^		1.8 ± 0.05x10^5^	
Pyrimethamine	1% EtOH	2.4 ± 0.8x10^5^	<0.01	7.0 ± 1.7x10^5^	0.01
	7.5 μM (10x)	9.3 ± 3.9x10^4^		1.0 ± 0.2x10^5^	
	0.5% EtOH	1.8 ± 0.1x10^5^	0.06	7.1 ± 0.6x10^5^	0.82
	3.7 μM (5x)	1.3 ± 0.1x10^5^		7.0 ± 0.7x10^5^	

^1^MCA: 3-dimensional multicellular aggregates.

^2^IC_50_: half maximal inhibitory concentration.

Ouabain is a cardiac glycoside that inhibits Na+/K + ATPase. The antiproliferative effect was validated in NCI-H295R monolayer cell cultures and MCA of both cell lines. At 0.08 μM and 0.8 μM (IC_50_ and 10 fold of IC_50_, respectively), ouabain demonstrated an antiproliferative effect in NCI-H295R cells with 57% and 89% growth inhibition, respectively. Both concentrations of ouabain also caused cell death by 13% and 77% (Figure 3B). At 0.08 μM (IC_50_), ouabain inhibited cellular proliferation of SW-13 cells with 62% cell death at day 4 (Figure 3E). At 0.08 μM, 0.16 μM and 0.8 μM (1 fold, 2 fold and 10 fold of IC50, respectively), ouabain inhibited growth and MCA in both cell lines were significantly smaller. Disintegration of MCA occurred in SW-13 MCA at all concentrations (Figure 4B and Table 3).

Methotrexate is a dihydrofolate Reductase (DHFR) inhibitor with antineoplastic and immunomodulator effects. The antiproliferative effect of methotrexate was validated only when NCI-H295R cells in monolayer culture were treated with a concentration of 0.2 μM (10 fold of IC_50_) (Figure 3C). At 0.2 μM (10 fold of IC_50_), methotrexate demonstrated antiproliferative effect in SW-13 cells (Figure 3E). However, Methotrexate has a very high therapeutic range (10 to 100 μM) and the maximal serum level achievable is over 500 fold of IC_50_ in the qHTS. Thus, we evaluated the antiproliferative effect of Methotrexate in MCA using concentrations of 0.2, 2 and 20 μM. Methotrexate inhibited the growth of MCA in both cell lines. Disintegration of NCI-H295R and SW-13 MCA occurred after 7 and 3 weeks of treatment, respectively (Figure 4C and Table 3).

Pyrimethamine is also a DHFR inhibitor, used in patients with protozoal infection such as toxoplasmosis. The antiproliferative effect of pyrimethamine in monolayer cultures of NCI-H295R cells was observed only when cells were treated with a concentration of 7.5 μM (10 fold of IC_50_) (Figure 3D). At 7.5 μM (10 fold of IC_50_), pyrimethamine demonstrated antiproliferative effect in SW-13 cells (Figure 3E). Since the maximal serum level of pyrimethamine is over 10 fold of IC_50_, we tested the effect of pyrimethamine in MCA of both cell lines using the concentrations of 3.7 and 7.5 μM (5 and 10 fold of IC_50_). Pyrimethamine caused growth inhibition, and resulted in significantly smaller and fewer MCA of NCI-H295R and SW-13 cells at 7.5 μM concentration (10 fold of IC_50_) but had no significant difference in size of MCA when either cell line was treated with 3.7 μM pyrimethamine (5 fold of IC_50_) (Figure 4D and Table 3).

### Effects of 4 candidate drugs on apoptosis

**Figure 5:**
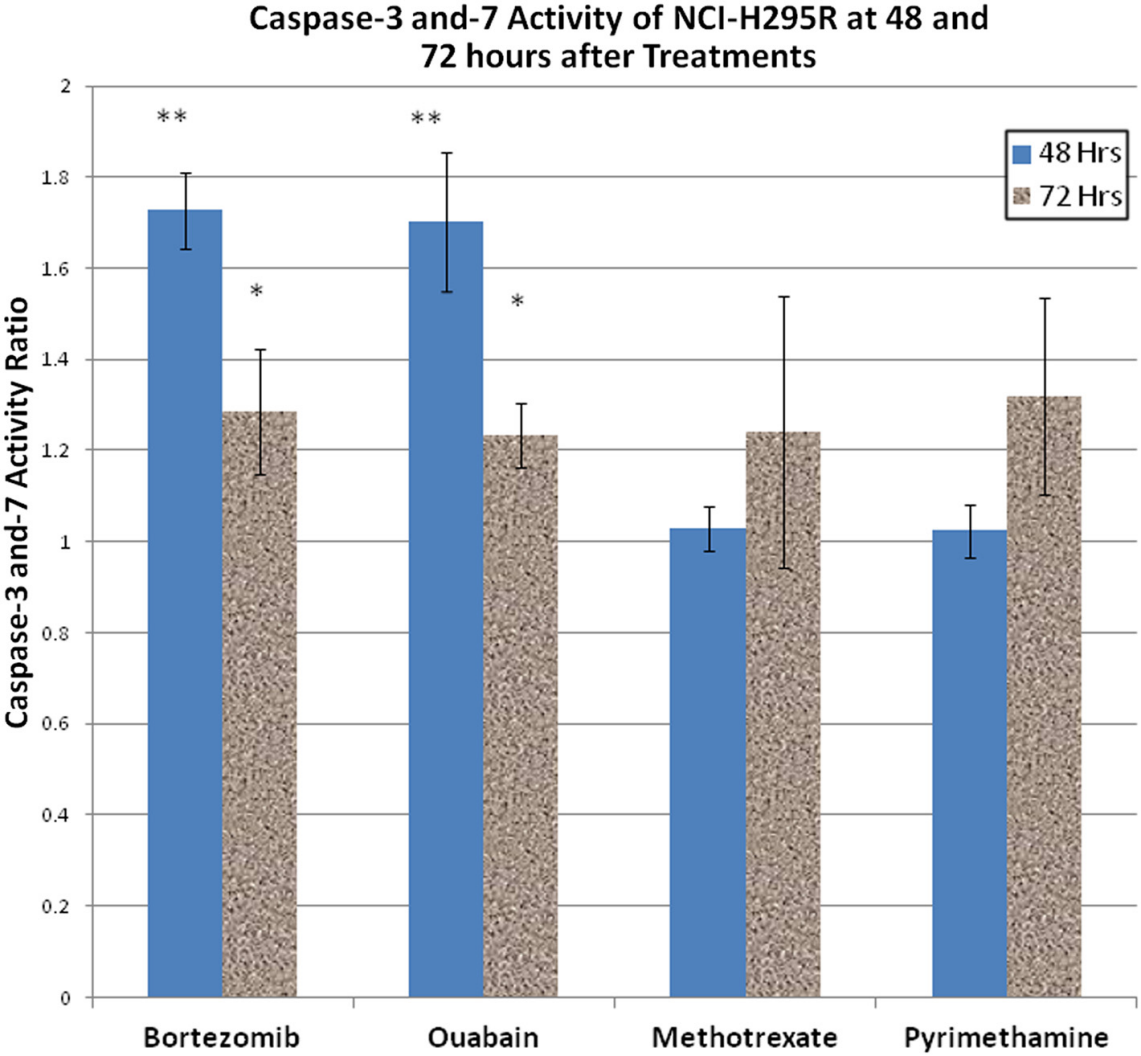
**Caspase-3 and -7 activity of NCI-H295R treated with bortezomib, ouabain, methotrexate, and pyrimethamine for 48 and 72 hours.** The results were shown in the ratios of caspase activity in treated NCI-H295R to corresponding vehicle controls. Caspase activities of treated cells were compared to respective vehicle controls. * *p* value <0.05 but ≥ 0.01, ** *p* value < 0.01.

Caspase-3 and -7 activities were significantly higher in NCI-H295R cells treated with 0.34 μM Bortezomib (*p* <0.01) and 0.08 μM ouabain (*p <* 0.01), compared to vehicle control groups, at 48 hours and 72 hours (*p =* 0.02 and *p =* 0.01, respectively) (Figure 5). We observed a lower caspase activity at 72 hours because most cells were dead by 72 hours. There was no significant difference in caspase activation when NCI-H295R cells were treated with methotrexate (*p =* 0.32) and pyrimethamine (*p =* 0.47) at 48 hours and 72 hours (*p =* 0.19 and *p =* 0.06, respectively) (Figure 5).

### Effects of methotrexate and pyrimethamine on cell cycle

**Figure 6:**
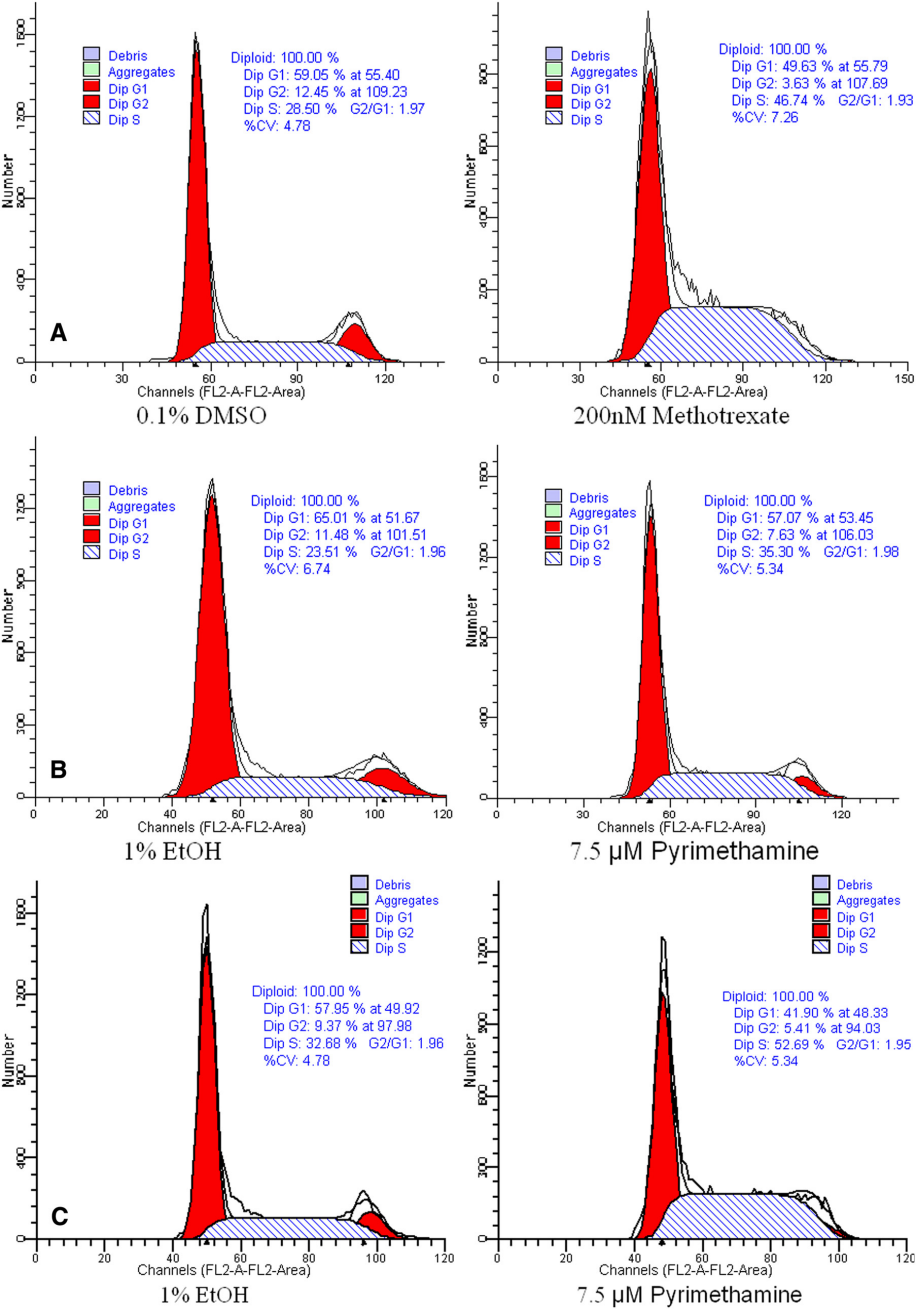
**Flow cytometry demonstrated that methotrexate caused S-phase cell cycle arrest in NCI-H295R (A) and pyrimethamine resulted in a modest increase of NCI-H295R cells in S-phase (B).** Pyrimethamine caused S-phase cell cycle arrest in SW-13 (**C**).

Increasing number of NCI-H295R cells in S-phase, from 28.5% to 46.7%, was observed when cells were treated with 0.2 μM (10 fold of IC_50_) methotrexate (Figure 6A). We observed a modest increase in number of NCI-H295R cells in S-phase, from 23.4% to 35.3%, when treated with pyrimethamine (Figure 6B). However, we found that 7.5 μM pyrimethamine caused an increase in number of SW-13 cells in S-phase, from 32.7% to 52.7% (Figure 6C).

## Discussion

Our study demonstrates an efficient and comprehensive approach to identify compounds with antiproliferative effect against ACC cells. We performed qHTS of a large, clinically approved, pharmaceutical collection with the goal to identify drugs that can be readily translated into at least a phase II clinical trial. Initially, qHTS was performed in NCI-H295R ACC cells which produce several corticosteroids as well as characteristic ACC phenotypes *in vivo*[20]*.* Of 21 high-confidence active compounds from various therapeutic categories that had efficacy greater than 60%; Bortezomib, ouabain, methotrexate and pyrimethamine were validated in monolayer cultures and MCA in both NCI-H295R and SW-13 cell lines. We believe that MCA model using two authenticated ACC cell lines used in this study better recapitulate *in vivo* ACC tumor growth than just monolayer. Several important characteristics of solid tumors, including the development of an extracellular matrix (ECM), cellular junctions between epithelial cells are observed in MCA. Similar to *in vivo* solid tumors, there are various concentrations of oxygen and nutrients as well as the different rates of cell proliferation from the outer layer to the center which can result in central necrosis and regions of hypoxia [21]. In addition, MCA have transcriptional profiles that are more representative of solid tumor, different than monolayer culture [22].

Bortezomib binds the catalytic site of 26S proteasome and causes apoptosis by upregulating NOXA, a proapoptotic protein and by suppressing nuclear factor kappa-light-chain-enhancer of activated B cells (NF-kB) signaling pathway [23]. There are numerous ongoing clinical trials using Bortezomib combined with various other agents in several types of solid tumors, some with promising initial results [24]. We have validated the antineoplastic effect of bortezomib in both NCI-H295R and SW-13 cell lines in monolayer culture and MCA. Our results also suggest that Bortezomib causes apoptosis in NCI-H295R cells.

Ouabain is a cardiac glycoside that inhibits cell membrane Na+/K + ATPase at high concentrations. In addition to an inotropic effect, cardiac glycosides have been explored for their antineoplastic effect [25-29]. Potential mechanisms for antineoplastic effect of cardiac glycosides include alteration of homeostasis of sodium, potassium and calcium, inhibition of topoisomerase II, glycolysis, and tumor necrosis factor/NF-kB pathways [29]. Although cardiac glycosides can cause cytotoxicity and inhibit protein synthesis in non-malignant cells [30], some studies suggest that cancer cells are more susceptible to the antiproliferative effect of cardiac glycosides [31,32]. The results from our qHTS showed that several cardiac glycosides active against ACC cells had excellent efficacy. However, most of these drugs had IC_50_ at serum concentrations which would be associated with toxicity in humans. In patients with breast cancer, an anticancer activity of digitalis has been observed [33-36], which is consistent with *in vitro* experiments that showed that the anticancer effects of cardiac glycosides in breast cancer cells can be achieved at the concentrations close or at therapeutic plasma levels [37-39]. We demonstrated that ouabain has a potent antiproliferative effect against NCI-H295R cells at clinically achievable concentration in both monolayer cultures and MCA, and causes apoptosis. A similar effect was observed in SW-13 MCA at IC_50_ and higher concentrations. Methotrexate and pyrimethamine are both DHFR inhibitors. Methotrexate has been used clinically as an anticancer drug and immunomodulator for over 50 years. The antineoplastic effect of pyrimethamine has been recently demonstrated in melanoma by inducing apoptosis [40,41]. On the other hand, pyrimethamine was reported to stimulate the growth of breast cancer cells (MCF-7) [42]. We did not find Methotrexate and pyrimethamine cause significant activation of caspase 3 and 7 or morphologic changes in monolayer culture. The antiproliferative effect of both DHFR inhibitors in ACC cells is likely due to a disruption of thymidylate and purine nucleotide biosynthesis during the S-phase of cell cycle [43]. The result from cell cycle analysis in NCI-H295R cells treated with methotrexate was consistent with S-phase cell cycle arrest as previously reported by others [44,45]. Similarly, we observed an increase of pyrimethamine treated SW-13 cells in S-phase, consistent with a disruption of DNA synthesis caused by DHFR inhibitor [40,46]. Both methotrexate and pyrimethamine had antiproliferative effect at 10 fold of IC_50_ concentration in monolayer cultures. The difference observed in MCA occurred after several weeks of exposure.

Since pyrimethamine and methotrexate have high therapeutic margins, a higher concentration (10 fold and 100 fold of IC_50_, respectively) can be administered. Growth inhibition of SW-13 and NCI-H295R MCA treated with high concentration of pyrimethamine (10 fold of IC_50_) was observed.

To our knowledge, this is the first study to use such a large collection of clinical drugs to test antiproliferative effect in ACC cells. These findings support the utility of qHTS of clinical drug library as a feasible approach for screening drug activity in other cancer cell lines from various other solid and hematologic malignancies. Because the costs and resources required for developing a new drug for rare cancers, such as ACC, are often prohibitive, the qHTS is an excellent, relatively inexpensive approach to identify effective agents in a short period of time. The discovery of new anticancer drugs using well-known compounds has several important implications. Patients with locally advanced and/or metastatic ACC could benefit from the identification of clinically approved agents that show anticancer effect specific to ACC cells and the prompt development of clinical trials to test the efficacy of these compounds can be initiated in relatively shorter time compared to the time required to bring unapproved compounds to clinical trials. An *in vivo* testing may still be necessary to identify the most effective drugs and or combination and to assess the different toxicity profile generated by drug combination treatment. Because drug toxicity is a common reason for discontinuation of therapy and poor compliance to the treatment, screening of existing drugs for new activity may be helpful because the toxicity of these drugs is well characterized and the most effective agents with the lowest toxicity profile can be selected. The toxicity may also be predicted and mitigated by using various countermeasures known for specific drugs. Furthermore, agents with clinical achievable concentrations can be determined after qHTS and the selection of those agents with potent activity well below the clinical sustained and peak concentrations of a drug is also a very attractive approach to use for cancer therapeutics. Even drugs that have IC_50_ above maximal serum level could possibly be administered locally or regionally, such as catheter-based treatment of metastasis confined to the liver, to reduce systemic toxicity.

In addition, many of the known drugs have well documented mechanism of actions which can be further explored to help understand tumor biology and pathways associated with tumor initiation and progression. The insight gained from altered molecular pathways may be used to create more effective drugs with less toxicity. Drug mechanism of action or therapeutic category enrichment analysis of active drugs can help identify mechanisms or pathways involved in tumor genesis. The common mechanism among the active drugs in our enrichment analysis warrants further investigation. We found 8 of 21 most effective drugs against NCI-H295R identified by qHTS were cardiac glycosides (Table 2). Our study is the first to describe *in vitro* antiproliferative effect of cardiac glycosides in ACC cells. Several other studies have reported *in vitro* and *in vivo* antiproliferative and apoptotic effects of these drugs in solid and hematologic cancer cell lines [29]. The enrichment analysis of active drugs can also be helpful in population-based study to evaluate cancer-risk reduction associated with specific drug use found to be active in our screening assay. Recently, guided by the results of their qHTS in prostate cancer cells, Platz, et al demonstrated that chronic Digoxin users have 25% lower risk of developing prostate cancer [8].

We have demonstrated that qHTS is an efficient method to identify active drugs against cancer cells; however, qHTS may not be able to identify drugs that may have activity after 48 hours of treatment. Because each well in the 1536-well plate contains only 5 μL, an incubation time longer than 48 hours would have significant evaporative loss and would result in inconsistent drug concentrations and high degree of variation.

One of the limitations in this study is not being able to generate primary cultures of human ACC with sufficient cell number and propagation to test. Only NCI-H295R cell line was screened with qHTS and screening multiple cell lines may identify additional active drugs. Furthermore, qHTS in multiple ACC cell lines can demonstrate the antineoplastic spectrum of the active compounds and a subset of high-efficacy compounds that are active across most ACC cell lines may be chosen for further validation. In summary, we have identified 79 active compounds from various therapeutic categories against ACC cell lines using a qHTS of clinical drug library screening approach. Four promising drugs (bortezomib, ouabain, methotrexate and pyrimethamine) were validated. The current study shows promising results in MCA treated with single candidate drug. Our results support the use of qHTS of clinical pharmaceutical libraries in different cancer types to identify drugs with potent activity that can be readily translated into clinical trials for patients with incurable malignancies or those with cancers refractory to standard therapy.

## Conclusions

qHTS of previously approved compounds is an effective strategy to identify candidate drugs with antineoplastic activity. We have validated the antineoplastic effect of bortezomib, ouabain, methotrexate and pyrimethamine in 2 ACC cell lines which can be translated into phase II clinical trials in patients with locally advanced and/or metastatic ACC.

## Competing interests

All authors declare that they have no competing interests.

## Authors’ contributions

NN designed, conducted *in vitro* validation of qHTS, analyzed data, performed statistical analysis and prepared the manuscript. MS, MH, YQZ, and CPA designed, conducted and analyzed the data of qHTS and edited the manuscript. LZ and EK involved in all processes of study design, interpretation and analysis of data as well as manuscript preparation. All authors read and approved the final manuscript.

## Supplementary Material

Additional file 1 The list of 2,816, clinically utilized, compounds used in quantitative high-throughput screening. Click here for file

Additional file 2 **Figure S1.** Quantitative high-throughput screening performance assessment. Figures show low plate variation, high signal to background (S/B) ratio, and Z-factor between 0.5 to 1.0, indicating excellent assay performance. Click here for file

Additional file 3 **References.** References of pharmacokinetics of 21 selected active drugs. Click here for file

Additional file 4 **Table S1.** Seventy-nine active compounds against NCI-H295R from quantitative high throughput screening. Click here for file
