# Supplementary figures and images for: Four clinically utilized drugs were identified and validated for treatment of adrenocortical cancer using quantitative high-throughput screening
Source: J Transl Med. 2012 Sep 21;10:198. doi: 10.1186/1479-5876-10-198 (PMC3493320; doi:10.1186/1479-5876-10-198)

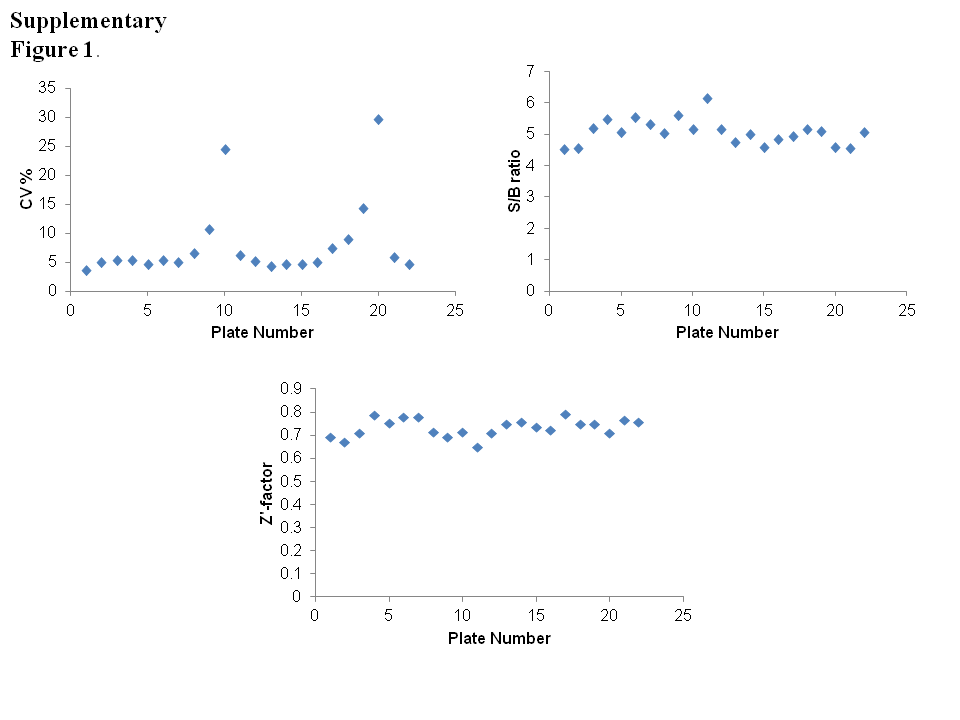

Supplement: Additional file 2 — Figure S1. Quantitative high-throughput screening performance assessment. Figures show low plate variation, high signal to background (S/B) ratio, and Z-factor between 0.5 to 1.0, indicating excellent assay performance. [file 1479-5876-10-198-S2.tiff]
