# Supplementary material for: Four clinically utilized drugs were identified and validated for treatment of adrenocortical cancer using quantitative high-throughput screening
Source: J Transl Med. 2012 Sep 21;10:198. doi: 10.1186/1479-5876-10-198 (PMC3493320; doi:10.1186/1479-5876-10-198)
Supplement: Additional file 3 — References. References of pharmacokinetics of 21 selected active drugs. [file 1479-5876-10-198-S3.docx]

Supplementary References:

| **Drug** | **Reference** |  |
| --- | --- | --- |
| ***Aclarubicin*** | Karanes C, Young JD, Samson MK, Smith LB, Franco LA, Baker LH: **Phase I trial of aclacinomycin-A. A clinical and pharmacokinetic study.** *Invest New Drugs* 1983, **1:**173-179 |  |
| ***Actinomycin D*** | Veal GJ, Cole M, Errington J, Parry A, Hale J, Pearson AD, Howe K, Chisholm JC, Beane C, Brennan B, et al: **Pharmacokinetics of dactinomycin in a pediatric patient population: a United Kingdom Children's Cancer Study Group Study.** *Clin Cancer Res* 2005, **11:**5893-5899. |  |
| ***Bortezomib*** | Leveque D, Carvalho MC, Maloisel F: **Review. Clinical pharmacokinetics of bortezomib.** *In Vivo* 2007, **21:**273-278. |  |
| ***Carboquone*** | Not available |  |
| ***Ciclopirox*** | Not available |  |
| ***Deslanoside*** | Bakke OM, Aslaksen A, Lehmann V, Lien E: **Pharmacokinetics and serum concentration--effect relationship of intravenous deslanoside.** *J Cardiovasc Pharmacol* 1981, **3:**1015-1025. |  |
| ***Digitoxin*** | Perrier D, Mayersohn M, Marcus FI: **Clinical pharmacokinetics of digitoxin.** *Clin Pharmacokinet* 1977, **2:**292-311. |  |
| ***Digoxin*** | Product Information: Lanoxin(R), digoxin. Glaxo Wellcome Inc, Research Triangle Park, NC, 98. |  |
| ***Ecteinascidin*** | Villalona-Calero MA, Eckhardt SG, Weiss G, Hidalgo M, Beijnen JH, van Kesteren C, Rosing H, Campbell E, Kraynak M, Lopez-Lazaro L, et al: **A phase I and pharmacokinetic study of ecteinascidin-743 on a daily x 5 schedule in patients with solid malignancies.** *Clin Cancer Res* 2002, **8:**75-85. |  |
|  | Ryan DP, Supko JG, Eder JP, Seiden MV, Demetri G, Lynch TJ, Fischman AJ, Davis J, Jimeno J, Clark JW: **Phase I and pharmacokinetic study of ecteinascidin 743 administered as a 72-hour continuous intravenous infusion in patients with solid malignancies.** *Clin Cancer Res* 2001, **7:**231-242. |  |
|  | van Kesteren C, Twelves C, Bowman A, Hoekman K, Lopez-Lazaro L, Jimeno J, Guzman C, Mathot RA, Simpson A, Vermorken JB, et al: **Clinical pharmacology of the novel marine-derived anticancer agent Ecteinascidin 743 administered as a 1- and 3-h infusion in a phase I study.** *Anticancer Drugs* 2002, **13:**381-393. |  |
| ***Homoharringtonine*** | Levy V, Zohar S, Bardin C, Vekhoff A, Chaoui D, Rio B, Legrand O, Sentenac S, Rousselot P, Raffoux E, et al: **A phase I dose-finding and pharmacokinetic study of subcutaneous semisynthetic homoharringtonine (ssHHT) in patients with advanced acute myeloid leukaemia.** *British journal of cancer* 2006, **95:**253-259 |  |
| ***Lanatoside A*** | Not available |  |
| ***Lanatoside C*** | Not available |  |
| ***Methotrexate*** | Bleyer WA: **The clinical pharmacology of methotrexate: new applications of an old drug.** *Cancer* 1978, **41:**36-51. |  |
| ***Metildigoxin*** | Not available |  |
| ***Niclosamide*** | Not available |  |
| ***Ouabain*** | Selden R, Smith TW: **Ouabain pharmacokinetics in dog and man. Determination by radioimmunoassay.** *Circulation* 1972, **45:**1176-1182.. |  |
| ***Proscillaridin A*** | Belz GG, Schreiter H, Wolf GK: **Pharmacokinetics and pharmacodynamics of methyl proscillaridin in healthy man.** *Eur J Clin Pharmacol* 1976, **10:**101-108. |  |
| ***Pyrimethamine*** | Jacobson JM, Davidian M, Rainey PM, Hafner R, Raasch RH, Luft BJ: **Pyrimethamine pharmacokinetics in human immunodeficiency virus-positive patients seropositive for Toxoplasma gondii.** *Antimicrob Agents Chemother* 1996, **40:**1360-1365. |  |
| ***Rotenone*** | Not available |  |
| ***Thioinosine*** | Not available |  |
| ***Trimetrexate glucuronate*** | Lin JT, Cashmore AR, Baker M, Dreyer RN, Ernstoff M, Marsh JC, Bertino JR, Whitfield LR, Delap R, Grillo-Lopez A: **Phase I studies with trimetrexate: clinical pharmacology, analytical methodology, and pharmacokinetics.** *Cancer research* 1987, **47:**609-616.Cancer Res 1987; 47:2996-2999. |  |
