# Supplementary material for: Four clinically utilized drugs were identified and validated for treatment of adrenocortical cancer using quantitative high-throughput screening
Source: J Transl Med. 2012 Sep 21;10:198. doi: 10.1186/1479-5876-10-198 (PMC3493320; doi:10.1186/1479-5876-10-198)
Supplement: Additional file 4 — Table S1. Seventy-nine active compounds against NCI-H295R from quantitative high throughput screening. [file 1479-5876-10-198-S4.doc]

**Supplementary Table 1.** Seventy-nine active compounds against NCI-H295R from quantitative high throughput screening.

| **Sample Name** | **Curve Class** | **IC50(uM)** | **Efficacy(%)** |
| --- | --- | --- | --- |
| Ecteinascidin 743 | -1.1 | 0.01 | -131 |
| Proscillaridin A | -1.1 | 0.01 | -131 |
| Trimetrexate glucuroante | -1.1 | 0.01 | -91 |
| Methotrexate | -1.2 | 0.02 | -71 |
| Digitoxin | -1.1 | 0.08 | -114 |
| Ouabain | -1.1 | 0.08 | -112 |
| Digoxin | -1.1 | 0.19 | -109 |
| Lanatoside A | -1.1 | 0.19 | -118 |
| Homoharringtonine | -1.1 | 0.21 | -116 |
| Metildigoxin | -1.1 | 0.24 | -112 |
| Lanatoside C | -1.1 | 0.30 | -127 |
| Bortezomib | -1.2 | 0.34 | -86 |
| Deslanoside | -1.1 | 0.34 | -114 |
| Actinomycin D | -1.1 | 0.38 | -114 |
| Niclosamide | -1.1 | 0.53 | -99 |
| Ciclopirox ethanolamine | -1.2 | 0.64 | -68 |
| Carboquone | -1.1 | 0.75 | -120 |
| Pyrimethamine | -1.1 | 0.75 | -82 |
| Rotenone | -1.2 | 0.75 | -70 |
| Aclarubicin | -1.1 | 0.94 | -121 |
| Plicamycin | -1.1 | 0.94 | -116 |
| 6-Thioguanine (6-TG) | -1.2 | 1.06 | -73 |
| Chromomycin A3 | -1.1 | 1.06 | -115 |
| Carminomycin | -1.1 | 1.19 | -124 |
| Cantharidin | -1.1 | 1.43 | -110 |
| Idarubicin HCL | -1.1 | 1.68 | -121 |
| Auranofin | -1.1 | 1.88 | -131 |
| Carubicinum | -1.1 | 1.88 | -121 |
| Tomatine | -1.1 | 1.88 | -129 |
| Emetine | -1.1 | 2.11 | -129 |
| 6-Mercaptopurine monohydrate | -1.2 | 2.37 | -66 |
| Quinocidum | -1.1 | 2.37 | -122 |
| Thimerosal H | -1.1 | 2.66 | -131 |
| Triamterene | -2.1 | 2.66 | -92 |
| Zinc pyrithione | -1.1 | 2.66 | -127 |
| Thionosine | -1.2 | 2.99 | -68 |
| Heliomycinum | -2.1 | 3.35 | -99 |
| Nitroxoline | -1.1 | 3.35 | -82 |
| Mitoxantrone dihydrochloride | -2.2 | 3.60 | -61 |
| Amsacrine hydrochloride | -2.2 | 3.76 | -63 |
| Phanquone | -2.2 | 3.76 | -65 |
| Cambendazole | -1.2 | 4.22 | -62 |
| Closantel | -2.1 | 4.22 | -129 |
| Ivermectin | -1.1 | 4.22 | -123 |
| Dienestrol | -2.2 | 4.53 | -72 |
| Rafoxanide | -2.1 | 4.73 | -137 |
| Sanguinarine | -1.1 | 4.73 | -117 |
| Chloroxine | -2.1 | 5.31 | -109 |
| Deslorelin acetate | -1.1 | 5.31 | -117 |
| Iodoquinol | -2.2 | 5.31 | -89 |
| Narasin | -2.1 | 5.31 | -137 |
| Nitrovin | -1.1 | 5.31 | -124 |
| Tribromsalan | -2.1 | 5.31 | -125 |
| Fluorosalan | -2.1 | 5.96 | -135 |
| Adapalene | -2.1 | 6.68 | -128 |
| Broxyquinoline | -1.1 | 6.68 | -82 |
| Bufogenin | -1.1 | 6.68 | -105 |
| Pyrvinium pamoate | -2.2 | 7.18 | -71 |
| Benziodarone | -2.1 | 7.50 | -99 |
| Hycanthone | -2.1 | 7.50 | -124 |
| Tannic acid | -2.1 | 7.50 | -124 |
| Tyrothricin | -2.1 | 7.50 | -130 |
| 17-Allylamino-geldanamycin | -2.1 | 8.41 | -104 |
| Aminopterin | -2.2 | 8.41 | -80 |
| Colestolone | -2.2 | 8.41 | -60 |
| Gramicidin | -2.1 | 8.41 | -124 |
| Iodochlorohydroxyquinoline | -2.2 | 8.41 | -61 |
| Olvanil | -2.1 | 8.41 | -123 |
| Teniposide | -2.1 | 8.41 | -97 |
| Boldo | -2.1 | 9.44 | -116 |
| Orlistat | -2.2 | 9.44 | -64 |
| Retinoic acid p-hydroxyanilide | -2.1 | 9.44 | -126 |
| Daunorubicinum | -2.1 | 10.59 | -126 |
| Gossypol | -2.1 | 10.59 | -129 |
| Mibefradil dihydrochloride | -2.1 | 10.59 | -119 |
| Nitroblue tetrazolium chrolide I | -2.1 | 10.59 | -130 |
| Sorafenib toyslate | -2.1 | 10.59 | -130 |
| Azathioprine | -2.2 | 10.59 | -74 |
| Doxorubicin | -2.1 | 10.59 | -100 |
